# Supplementary material for: Alteration of Serum Proteome in Levo-Thyroxine-Euthyroid Thyroidectomized Patients
Source: J Clin Med. 2022 Mar 17;11(6):1676. doi: 10.3390/jcm11061676 (PMC8951767; doi:10.3390/jcm11061676)
Supplement: Supplementary file 1 [file jcm-11-01676-s001.zip › jcm-1572550-supplementary.pdf]

Supplementary Tables

Table S1. Canonical paths reported in the protein network in Figure 6A (edges highlighted in light blue). GO biological processes are reported for each canonical path together with p-values.

	Network	GO processes	p-Value
1	Albumin, NANOG, c-Myc, SOX2, GATA-4	response to organic substance (93.3%; 1.673×10^{-17}), response to endogenous stimulus (73.3%; 1.672×10^{-15}), formation of primary germ layer (33.3%; 2.911×10^{-14}), gastrulation (36.7%; 2.933×10^{-14}), response to oxygen-containing compound (73.3%; 3.298×10^{-14})	1.350×10^{-48}
2	Alpha1-globin, MSP receptor (RON), SHP-1, Epo, MGF	response to organic substance (93.6%; 4.855×10^{-27}), cytokine-mediated signaling pathway (57.4%; 2.429×10^{-26}), positive regulation of response to stimulus (80.9%; 4.689×10^{-25}), cellular response to cytokine stimulus (63.8%; 1.844×10^{-24}), regulation of immune system process (72.3%; 2.640×10^{-24})	4.480×10^{-45}
3	C3c, NF-kB, alpha-M/beta-2 integrin, Adenosine A2a receptor, alpha-X/beta-2 integrin	activation of immune response (75.8%; 1.439×10^{-31}), immune effector process (87.9%; 5.158×10^{-31}), complement activation (57.6%; 5.556×10^{-31}), positive regulation of immune response (81.8%; 7.722×10^{-31}), regulation of immune response (84.8%; 4.929×10^{-29})	8.710×10^{-45}
4	APOE, PLTP, S1P3 receptor, S1P1 receptor, Chylomicron remnant	chylomicron remodeling (31.4%; 9.603×10^{-36}), plasma lipoprotein particle remodeling (35.3%; 1.030×10^{-35}), protein-lipid complex remodeling (35.3%; 1.030×10^{-35}), plasma lipoprotein particle organization (37.3%; 5.243×10^{-35}), protein-containing complex remodeling (35.3%; 6.099×10^{-35})	3×10^{-36}
5	Factor B, C3c, C5aR, C3aR, alpha-M/beta-2 integrin	regulation of complement activation (58.3%; 1.720×10^{-26}), regulation of humoral immune response (58.3%; 7.029×10^{-25}), complement activation, alternative pathway (33.3%; 7.470×10^{-21}), regulation of immune effector process (70.8%; 8.496×10^{-21}), positive regulation of immune system process (83.3%; 2.145×10^{-20})	6.890×10^{-35}
6	Factor B, C3c, alpha-M/beta-2 integrin, C5aR, C5b	leukocyte mediated immunity (90.0%; 5.537×10^{-12}), complement activation, alternative pathway (40.0%; 3.860×10^{-11}), complement receptor mediated signaling pathway (40.0%; 4.962×10^{-11}), immune effector process (90.0%; 1.592×10^{-10}), regulated exocytosis (80.0%; 1.995×10^{-10})	2.060×10^{-34}
7	Angiostatin, Plasmin, RelA (p65 NF-kB subunit), PPAR-gamma, PLAU (UPA)	response to stress (100.0%; 1.409×10^{-12}), regulation of response to stress (77.8%; 1.519×10^{-12}), regeneration (50.0%; 5.246×10^{-12}), fibrinolysis (27.8%; 5.925×10^{-12}), negative regulation of blood coagulation (33.3%; 2.828×10^{-11})	6.350×10^{-35}
8	APOA1, SR-BI, S1P3 receptor, S1P1 receptor, Pre beta-1 HDL lipids extracellular region	plasma lipoprotein particle remodeling (44.8%; 1.436×10^{-27}), protein-lipid complex remodeling (44.8%; 1.436×10^{-27}), high-density lipoprotein particle remodeling (41.4%; 1.525×10^{-27}), organic hydroxy compound transport (58.6%; 4.048×10^{-27}), protein-containing complex remodeling (44.8%; 4.859×10^{-27})	2.360×10^{-32}
9	IL-1 beta, C3aR, PI3K cat class IB (p110-gamma), CCL5, IL-8	cell activation (71.8%; 1.522×10^{-24}), regulation of immune system process (79.5%; 2.008×10^{-24}), defense response (74.4%; 3.194×10^{-22}), positive regulation of response to stimulus (82.1%; 1.137×10^{-21}), immune response (76.9%; 1.188×10^{-21})	2.300×10^{-30}
10	C3c, alpha-M/beta-2 integrin, CR1, C3aR, C5aR	regulation of complement activation (66.7%; 4.525×10^{-20}), complement activation, alternative pathway (46.7%; 1.546×10^{-19}), regulation of humoral immune response (66.7%; 6.181×10^{-19}), leukocyte mediated immunity (93.3%; 1.274×10^{-18}), immune effector process (93.3%; 2.414×10^{-16})	1.590×10^{-30}

	Network	GO processes	p-Value
11	Pre beta-1 HDL lipids extracellular region, SR-BI, S1P1 receptor, S1P3 receptor, APOE	protein-lipid complex remodeling (85.7%; 1.034 × 10^{-30}), plasma lipoprotein particle remodeling (85.7%; 1.034 × 10^{-30}), protein-containing complex remodeling (85.7%; 3.157 × 10^{-30}), high-density lipoprotein particle remodeling (78.6%; 9.833 × 10^{-30}), plasma lipoprotein particle organization (85.7%; 8.793 × 10^{-29})	2.510 × 10^{-20}
12	C3c, Factor H, C3b, iC3b, Factor Bb	complement activation (100.0%; 2.778 × 10^{-23}), humoral immune response (100.0%; 1.271 × 10^{-18}), activation of immune response (100.0%; 2.380 × 10^{-17}), positive regulation of immune response (100.0%; 2.243 × 10^{-15}), innate immune response (100.0%; 8.922 × 10^{-15})	2.720 × 10^{-19}
13	CD21, iC3b, Factor H, C3b, Factor Bb	complement activation (100.0%; 2.402 × 10^{-25}), humoral immune response (100.0%; 2.969 × 10^{-20}), activation of immune response (100.0%; 7.277 × 10^{-19}), positive regulation of immune response (100.0%; 1.039 × 10^{-16}), regulation of complement activation (66.7%; 2.919 × 10^{-16})	3.580 × 10^{-19}
14	Factor B, C3b, Factor Bb, C5 convertase (C3b2Bb), Factor D	complement activation, alternative pathway (100.0%; 4.886 × 10-07), complement activation (100.0%; 8.264 × 10-05), humoral immune response (100.0%; 5.663 × 10-04), activation of immune response (100.0%; 9.611 × 10-04), leukocyte mediated immunity (100.0%; 1.919 × 10-03)	6.260 × 10^{-15}
15	S1P1 receptor, S1P3 receptor, APOE, APOA1, Pre beta-1 HDL	protein-lipid complex remodeling (81.8%; 7.902 × 10-23), plasma lipoprotein particle remodeling (81.8%; 7.902 × 10-23), protein-containing complex remodeling (81.8%; 1.781 × 10-22), plasma lipoprotein particle organization (81.8%; 2.033 × 10-21), high-density lipoprotein particle remodeling (72.7%; 2.667 × 10-21)	8.850 × 10^{-14}
16	HP, IL-6, STAT3, IL-6 receptor, IL6RA	interleukin-6-mediated signaling pathway (58.3%; 8.145 × 10-18), interleukin-27-mediated signaling pathway (50.0%; 2.868 × 10-16), cellular response to interleukin-6 (58.3%; 1.923 × 10-15), response to interleukin-6 (58.3%; 4.380 × 10-15), interleukin-35-mediated signaling pathway (41.7%; 5.499 × 10-13)	3.540 × 10^{-10}
17	Transferrin, HSP70, TCP1, HIF1A, VHL	iris morphogenesis (44.4%; 9.751 × 10-12), negative regulation of thymocyte apoptotic process (44.4%; 1.329 × 10-11), regulation of thymocyte apoptotic process (44.4%; 1.033 × 10-10), regulation of cellular response to hypoxia (44.4%; 1.453 × 10-10), negative regulation of T cell apoptotic process (44.4%; 5.082 × 10-10)	1.350 × 10^{-10}
18	Pre beta-1 HDL lipids extracellular region, Pre beta-1 HDL lipids + Cholesterol + Phospholipid = HDL nascent lipids, HDL nascent lipids extracellular region, Cholesterol extracellular region	cholesterol import (100.0%; 2.078 × 10-10), sterol import (100.0%; 2.078 × 10-10), negative regulation of cell adhesion molecule production (100.0%; 2.558 × 10-10), negative regulation of heterotypic cell-cell adhesion (100.0%; 5.207 × 10-10), regulation of cell-cell adhesion involved in gastrulation (100.0%; 5.207 × 10-10)	6.440 × 10^{-12}
19	SAR1A, Sec23, Chylomicron remnant, APOA4, VAMP7	chylomicron remodeling (60.0%; 2.238 × 10-23), triglyceride-rich lipoprotein particle remodeling (60.0%; 5.631 × 10-23), protein-lipid complex remodeling (60.0%; 7.138 × 10-21), plasma lipoprotein particle remodeling (60.0%; 7.138 × 10-21), regulation of plasma lipoprotein particle levels (66.7%; 1.491 × 10-20)	1.920 × 10^{-06}
20	Elastin, IL-17, CCL20, MIG, IP10	leukocyte migration (72.7%; 9.562 × 10-12), cell migration (81.8%; 3.687 × 10-10), inflammatory response (72.7%; 3.817 × 10-10), localization of cell (81.8%; 1.266 × 10-09), cell motility (81.8%; 1.266 × 10-09)	1.320 × 10^{-03}

Table S2. GO biological processes enrichment analysis by MetaCore software. Table reports p-values, FDR and network objects from active data.

	Networks	p-value	FDR	Network Objects from Active Data
1	Inflammation_Complement system	1.591×10^{-14}	3.818×10^{-13}	Factor H, C3, Factor Bb, C3dg, C3 convertase (C3bBb), C5 convertase (C3b2Bb), C3b, iC3b, C3a, Factor B
2	Inflammation_IL-6 signaling	2.032×10^{-06}	2.438×10^{-05}	C3, HP/HB complex, HDL proteins, HP, Alpha1-globin, Alpha 1-antitrypsin
3	Inflammation_Kallikrein-kinin system	2.761×10^{-05}	2.209×10^{-04}	Plasmin, C3, Plasminogen, Angiostatin, Alpha 1-antitrypsin, C3a
4	Immune response_Phagocytosis	7.433×10^{-05}	4.460×10^{-04}	C3, C3dg, HDL proteins, APOE, C3b, iC3b
5	Inflammation_Innate inflammatory response	2.891×10^{-03}	1.388×10^{-02}	C3, C5 convertase (C3b2Bb), C3b, C3a
6	Blood coagulation	3.588×10^{-03}	1.435×10^{-02}	Plasmin, Plasminogen, Alpha 1-antitrypsin
7	Transport_Iron transport	5.461×10^{-03}	1.872×10^{-02}	Apotransferrin, Holotransferrin, Transferrin
8	Proteolysis_Connective tissue degradation	6.984×10^{-03}	2.095×10^{-02}	Plasmin, Plasminogen, Alpha 1-antitrypsin
9	Cell adhesion_Platelet-endothelium-leucocyte interactions	1.996×10^{-02}	5.322×10^{-02}	Plasmin, Plasminogen, Angiostatin
10	Proteolysis_ECM remodeling	3.285×10^{-02}	7.884×10^{-02}	Plasmin, Alpha 1-antitrypsin

Table S3. Pathway Maps by MetaCore software reporting p-values, FDR and network objects from active data.

	Maps	p-value	FDR	Network Objects from Active Data
1	Immune response_Alternative complement pathway	1.398×10^{-21}	1.147×10^{-19}	Factor H, Factor Ba, C3c, C3, Factor Bb, C3dg, C3 convertase (C3bBb), C3b, C5 convertase (C3b2Bb), iC3b, C3a, Factor B
2	Alternative complement cascade disruption in age-related macular degeneration	8.608×10^{-20}	3.529×10^{-18}	Factor H, Factor Ba, C3, Factor Bb, C3 convertase (C3bBb), C3b, C5 convertase (C3b2Bb), iC3b, C3a, Factor B
3	Complement pathway disruption in thrombotic microangiopathy	4.676×10^{-10}	1.278×10^{-08}	Factor H, C3, C3 convertase (C3bBb), C3b, C5 convertase (C3b2Bb), C3a
4	Transport_HDL-mediated reverse cholesterol transport	7.478×10^{-10}	1.533×10^{-08}	HDL, Pre beta-1 HDL, APOE, APOA1, Large apoE-rich HDL, Nascent HDL
5	Immune response_Lectin induced complement pathway	2.234×10^{-09}	3.664×10^{-08}	C3c, C3, C3dg, C3b, iC3b, C3a
6	Immune response_Classical complement pathway	3.211×10^{-09}	4.388×10^{-08}	C3c, C3, C3dg, C3b, iC3b, C3a
7	HDL dyslipidemia in type 2 diabetes and metabolic syndrome X	3.405×10^{-08}	3.989×10^{-07}	HDL, Pre beta-1 HDL, APOE, APOA1, Nascent HDL
8	Lipoprotein metabolism	6.707×10^{-07}	6.875×10^{-06}	Pre beta-1 HDL, APOE, APOA1, APOA4, Nascent HDL
9	Role of ZNF202 in regulation of expression of genes involved in atherosclerosis	1.861×10^{-05}	1.695×10^{-04}	HDL proteins, APOE, APOA4
10	Plasminogen activators signaling in pancreatic cancer	8.941×10^{-05}	7.331×10^{-04}	Plasmin, Plasminogen, Angiostatin
11	Blood coagulation_Blood coagulation	1.240×10^{-04}	9.243×10^{-04}	Plasmin, Plasminogen, Alpha 1-antitrypsin

Figure S1. Mascot Search Results of spots 34, 35, 39, 44, 45, 50, 43.

Spot 34

MATRIX SCIENCE MASCOT Search Results

Protein View: APOA1_HUMAN

Apolipoprotein A-I OS=Homo sapiens OX=9606 GN=APOA1 PE=1 SV=1

**Database:**	SwissProt
**Score:**	428
**Expect:**	3.2e-39
**Monoisotopic mass (M_r):**	30759
**Calculated pI:**	5.56
**Taxonomy:**	Homo sapiens

Sequence similarity is available as an NCBI BLAST search of APOA1_HUMAN against nr.

Search parameters

**Enzyme:**	Trypsin: cuts C-term side of KR unless next residue is P.
**Fixed modifications:**	Carbamidomethyl (C)
**Variable modifications:**	Oxidation (M)
**Mass values searched:**	37
**Mass values matched:**	31

Protein sequence coverage: 74%

Matched peptides shown in ***bold red***.

```
  1 MKAAVLTLAV LFLTGSQARH FWQQDEPPQS PWDRVKDLAT VYVDVLKDSG
 51 RDYVSQFEGS ALGKQLNLKL LDNWDSVTST FSKLREQLGP VTQEFWDNLE
101 KETEGLRQEM SKDLEEVKAK VQPYLDDFQK KWQEEMELYR QKVEPLRAEL
151 QEGARQKLHE LQEKLSPLGE EMRDRARAHV DALRTHLAPY SDELRQRLAA
201 RLEALKENGG ARLAEYHAKA TEHLSTLSEK AKPALEDLRQ GLLPVLESFK
251 VSFLSALEEY TKKLNTQ
```

Unformatted sequence string: 267 residues (for pasting into other applications).

Sort by ◉ residue number ○ increasing mass ○ decreasing mass
Show ◉ matched peptides only ○ predicted peptides also

Start – End	Observed	Mr(expt)	Mr(calc)	ppm	M	Peptide
35 – 47	1462.8690	1461.8617	1461.8443	11.9	1	R.VKDLATVYVDVLK.D
37 – 47	1235.6710	1234.6637	1234.6809	-13.9	0	K.DLATVYVDVLK.D
37 – 51	1650.8710	1649.8637	1649.8625	0.77	1	K.DLATVYVDVLKDSGR.D
48 – 64	1815.8390	1814.8317	1814.8435	-6.49	1	K.DSGRDYVSQFEGSALGK.Q
52 – 64	1400.7000	1399.6927	1399.6620	22.0	0	R.DYVSQFEGSALGK.Q
70 – 83	1612.7990	1611.7917	1611.7781	8.48	0	K.LLDNWDSVTSTFSK.L
84 – 101	2202.1180	2201.1107	2201.1117	-0.43	1	K.LREQLGPVTQEFWDNLEK.E
86 – 101	1932.9400	1931.9327	1931.9265	3.22	0	R.EQLGPVTQEFWDNLEK.E
86 – 107	2618.3170	2617.3097	2617.2660	16.7	1	R.EQLGPVTQEFWDNLEKETEGLR.Q
102 – 107	704.3410	703.3337	703.3501	-23.2	0	K.ETEGLR.Q
102 – 112	1323.6030	1322.5957	1322.6136	-13.5	1	K.ETEGLRQEMSK.D + Oxidation (M)
121 – 130	1252.6380	1251.6307	1251.6136	13.7	0	K.VQPYLDDFQK.K
121 – 131	1380.7250	1379.7177	1379.7085	6.68	1	K.VQPYLDDFQKK.W
131 – 140	1411.6740	1410.6667	1410.6601	4.67	1	K.KWQEEMELYR.Q
131 – 140	1427.6950	1426.6877	1426.6551	22.9	1	K.KWQEEMELYR.Q + Oxidation (M)
132 – 140	1283.5760	1282.5687	1282.5652	2.76	0	K.WQEEMELYR.Q
132 – 140	1299.5860	1298.5787	1298.5601	14.3	0	K.WQEEMELYR.Q + Oxidation (M)
141 – 147	869.5240	868.5167	868.5130	4.25	1	R.QKVEPLR.A
143 – 155	1467.8010	1466.7937	1466.7841	6.56	1	K.VEPLRAELQEGAR.Q
148 – 155	873.4230	872.4157	872.4352	-22.3	0	R.AELQEGAR.Q
156 – 164	1152.6260	1151.6187	1151.6298	-9.65	1	R.QKLHELQEK.L
158 – 164	896.4850	895.4777	895.4763	1.59	0	K.LHELQEK.L
165 – 173	1031.5160	1030.5087	1030.5117	-2.89	0	K.LSPLGEEMR.D
165 – 173	1047.5100	1046.5027	1046.5066	-3.72	0	K.LSPLGEEMR.D + Oxidation (M)
178 – 184	781.4410	780.4337	780.4242	12.2	0	R.AHVDALR.T
185 – 195	1301.6580	1300.6507	1300.6411	7.37	0	R.THLAPYSDELR.Q
202 – 212	1157.6020	1156.5947	1156.6200	-21.8	1	R.LEALKENGGAR.L
220 – 230	1215.6010	1214.5937	1214.6143	-16.9	0	K.ATEHLSTLSEK.A
231 – 239	1012.5760	1011.5687	1011.5713	-2.51	0	K.AKPALEDLR.Q
240 – 250	1230.7230	1229.7157	1229.7020	11.2	0	R.QGLLPVLESFK.V
251 – 262	1386.7420	1385.7347	1385.7078	19.4	0	K.VSFLSALEEYTK.K

No match to: 1024.5740, 1170.6080, 1226.5520, 1662.8430, 1797.8870, 2630.3070

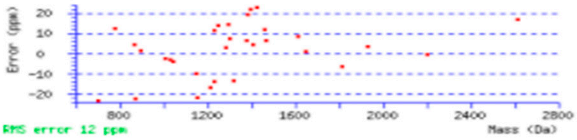

Spot 35

MATRIX SCIENCE MASCOT Search Results

Protein View: APOA1_HUMAN

Apolipoprotein A-I OS=Homo sapiens OX=9606 GN=APOA1 PE=1 SV=1

**Database:**	SwissProt
**Score:**	321
**Expect:**	1.6e-28
**Monoisotopic mass (M_r):**	30759
**Calculated pI:**	5.56
**Taxonomy:**	Homo sapiens

Sequence similarity is available as an NCBI BLAST search of APOA1_HUMAN against nr.

Search parameters

**Enzyme:**	Trypsin: cuts C-term side of KR unless next residue is P.
**Fixed modifications:**	Carbamidomethyl (C)
**Variable modifications:**	Oxidation (M)
**Mass values searched:**	34
**Mass values matched:**	26

Protein sequence coverage: 70%

Matched peptides shown in ***bold red***.

```
  1 MKAAVLTLAV LFLTGSQARH FWQQDEPPQS PWDRVKDLAT VYVDVLKDSG
 51 RDYVSQFEGS ALGKQLNLKL LDNWDSVTST FSKLREQLGP VTQEFWDNLE
101 KETEGLRQEM SKDLEEVKAK VQPYLDDFQK KWQEEMELYR QKVEPLRAEL
151 QEGARQKLHE LQEKLSPLGE EMRDRARAHV DALRTHLAPY SDELRQRLAA
201 RLEALKENGG ARLAEYHAKA TEHLSTLSEK AKPALEDLRQ GLLPVLESFK
251 VSFLSALEEY TKKLNTQ
```

Unformatted sequence string: 267 residues (for pasting into other applications).

Sort by ◉ residue number ○ increasing mass ○ decreasing mass
Show ◉ matched peptides only ○ predicted peptides also

Start – End	Observed	Mr(expt)	Mr(calc)	ppm	M	Peptide
20 – 34	1952.8360	1951.8287	1951.8602	-16.1	0	R.HFWQQDEPPQSPWDR.V
35 – 47	1462.8250	1461.8177	1461.8443	-18.2	1	R.VKDLATVYVDVLK.D
37 – 47	1235.6410	1234.6337	1234.6809	-38.2	0	K.DLATVYVDVLK.D
48 – 64	1815.8310	1814.8237	1814.8435	-10.9	1	K.DSGRDYVSQFEGSALGK.Q
52 – 64	1400.6650	1399.6577	1399.6620	-3.02	0	R.DYVSQFEGSALGK.Q
70 – 83	1612.7670	1611.7597	1611.7781	-11.4	0	K.LLDNWDSVTSTFSK.L
84 – 101	2202.1070	2201.0997	2201.1117	-5.42	1	K.LREQLGPVTQEFWDNLEK.E
86 – 101	1932.9080	1931.9007	1931.9265	-13.3	0	R.EQLGPVTQEFWDNLEK.E
86 – 107	2618.2820	2617.2747	2617.2660	3.34	1	R.EQLGPVTQEFWDNLEKETEGLR.Q
121 – 130	1252.6170	1251.6097	1251.6136	-3.06	0	K.VQPYLDDFQK.K
131 – 140	1411.6070	1410.5997	1410.6601	-42.8	1	K.KWQEEMELYR.Q
131 – 140	1427.6570	1426.6497	1426.6551	-3.74	1	K.KWQEEMELYR.Q + Oxidation (M)
132 – 140	1283.5520	1282.5447	1282.5652	-16.0	0	K.WQEEMELYR.Q
132 – 140	1299.5640	1298.5567	1298.5601	-2.60	0	K.WQEEMELYR.Q + Oxidation (M)
143 – 155	1467.7690	1466.7617	1466.7841	-15.3	1	K.VEPLRAELQEGAR.Q
148 – 155	873.4350	872.4277	872.4352	-8.53	0	R.AELQEGAR.Q
165 – 173	1031.5090	1030.5017	1030.5117	-9.68	0	K.LSPLGEEMR.D
165 – 173	1047.5130	1046.5057	1046.5066	-0.86	0	K.LSPLGEEMR.D + Oxidation (M)
165 – 175	1302.6270	1301.6197	1301.6398	-15.4	1	K.LSPLGEEMRDR.A
165 – 175	1318.6220	1317.6147	1317.6347	-15.1	1	K.LSPLGEEMRDR.A + Oxidation (M)
178 – 184	781.4380	780.4307	780.4242	8.33	0	R.AHVDALR.T
185 – 195	1301.6310	1300.6237	1300.6411	-13.4	0	R.THLAPYSDELR.Q
202 – 212	1157.6050	1156.5977	1156.6200	-19.3	1	R.LEALKENGGAR.L
231 – 239	1012.5760	1011.5687	1011.5713	-2.51	0	K.AKPALEDLR.Q
240 – 250	1230.7050	1229.6977	1229.7020	-3.45	0	R.QGLLPVLESFK.V
251 – 262	1386.6910	1385.6837	1385.7078	-17.4	0	K.VSFLSALEEYTK.K

No match to: 1054.9640, 1693.8230, 1723.9210, 1797.8750, 1811.8790, 1875.9150, 2630.2700, 2887.5200

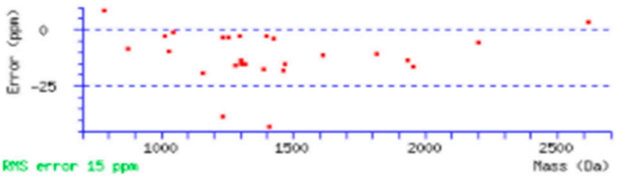

Spot 45

MATRIX SCIENCE Mascot Search Results

```
User            : Claudia Landi
Email           : landi35@unisi.it
Search title    : Siero Endocrinologia
Database        : SwissProt 2021_03 (565254 sequences; 203850821 residues)
Taxonomy        : Homo sapiens (human) (20387 sequences)
Timestamp       : 1 Sep 2021 at 09:51:19 GMT
Top Score       : 264 for Mixture 1, A1AT_HUMAN + APOA1_HUMAN
```

Mascot Score Histogram

Protein score is -10*Log(P), where P is the probability that the observed match is a random event.
Protein scores greater than 56 are significant (p<0.05).

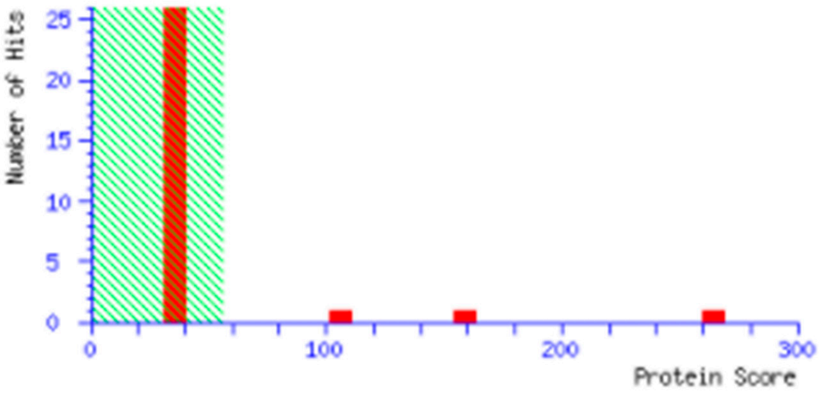

Concise Protein Summary Report

Format As | Concise Protein Summary | Help

Significance threshold p< 0.05 | Max. number of hits AUTO

Re-Search All | Search Unmatched

```
1.      Mixture 1     Total score: 264     Expect: 8.1e-23   Matches: 26
        Components (only one family member shown for each component):
        A1AT_HUMAN     Mass: 46878     Score: 165     Expect: 6.4e-13   Matches: 16
        Alpha-1-antitrypsin OS=Homo sapiens OX=9606 GN=SERPINA1 PE=1 SV=3
        APOA1_HUMAN     Mass: 30759     Score: 112     Expect: 1.3e-07   Matches: 11
        Apolipoprotein A-I OS=Homo sapiens OX=9606 GN=APOA1 PE=1 SV=1
```

MATRIX SCIENCE MASCOT Search Results

Protein View: A1AT_HUMAN

Alpha-1-antitrypsin OS=Homo sapiens OX=9606 GN=SERPINA1 PE=1 SV=3

**Database:**	SwissProt
**Score:**	165
**Expect:**	6.4e-13
**Monoisotopic mass (M_r):**	46878
**Calculated pI:**	5.37
**Taxonomy:**	Homo sapiens

Sequence similarity is available as an NCBI BLAST search of A1AT_HUMAN against nr.

Search parameters

**Enzyme:**	Trypsin: cuts C-term side of KR unless next residue is P.
**Fixed modifications:**	Carbamidomethyl (C)
**Variable modifications:**	Oxidation (M)
**Mass values searched:**	32
**Mass values matched:**	16

Protein sequence coverage: 34%

Matched peptides shown in ***bold red***.

```
  1 MPSSVSWGIL LLAGLCCLVP VSLAEDPQGD AAQKTDTSHH DQDHPTFNKI
 51 TPNLAEFAFS LYRQLAHQSN STNIFFSPVS IATAFAMLSL GTKADTHDEI
101 LEGLNFNLTE IPEAQIHEGF QELLRTLNQP DSQLQLTTGN GLFLSEGLKL
151 VDKFLEDVKK LYHSEAFTVN FGDTEEAKKQ INDYVEKGTQ GKIVDLVKEL
201 DRDTVFALVN YIFFKGKWER PFEVKDTEEE DFHVDQVTTV KVPMMKRLGM
251 FNIQHCKKLS SWVLLMKYLG NATAIFFLPD EGKLQHLENE LTHDIITKFL
301 ENEDRRSASL HLPKLSITGT YDLKSVLGQL GITKVFSNGA DLSGVTEEAP
351 LKLSKAVHKA VLTIDEKGTE AAGAMFLEAI PMSIPPEVKF NKPFVFLMIE
401 QNTKSPLFMG KVVNPTQK
```

Unformatted sequence string: 418 residues (for pasting into other applications).

Sort by ◉ residue number ○ increasing mass ○ decreasing mass
Show ◉ matched peptides only ○ predicted peptides also

Start - End	Observed	Mr(expt)	Mr(calc)	ppm	M	Peptide
126 - 149	2574.3410	2573.3337	2573.3337	0.012	0	R.TLNQPDSQLQLTTGNGLFLSEGLK.L
150 - 159	1205.6650	1204.6577	1204.6703	-10.5	1	K.LVDKFLEDVK.K
161 - 178	2057.9630	2056.9557	2056.9378	8.72	0	K.LYHSEAFTVNFGDTEEAK.K
161 - 179	2186.0410	2185.0337	2185.0327	0.45	1	K.LYHSEAFTVNFGDTEEAKK.Q
180 - 187	1008.4940	1007.4867	1007.4924	-5.59	0	K.QINDYVEK.G
199 - 215	2090.0920	2089.0847	2089.0884	-1.77	1	K.ELDRDTVFALVNYIFFK.G
203 - 215	1576.8160	1575.8087	1575.8337	-15.9	0	R.DTVFALVNYIFFK.G
216 - 225	1275.6930	1274.6857	1274.6771	6.74	1	K.GKWERPFEVK.D
218 - 225	1090.5770	1089.5697	1089.5607	8.27	0	K.WERPFEVK.D
218 - 241	2963.4160	2962.4087	2962.3985	3.45	1	K.WERPFEVKDTEEEDFHVDQVTTVK.V
226 - 241	1891.8490	1890.8417	1890.8483	-3.50	0	K.DTEEEDFHVDQVTTVK.V
248 - 257	1247.6020	1246.5947	1246.5951	-0.28	0	R.LGMFNIQHCK.K
248 - 257	1263.6060	1262.5987	1262.5900	6.91	0	R.LGMFNIQHCK.K + Oxidation (M)
259 - 267	1076.6120	1075.6047	1075.6100	-4.88	0	K.LSSWVLLMK.Y
284 - 298	1803.9640	1802.9567	1802.9526	2.27	0	K.LQHLENELTHDIITK.F
299 - 305	922.4340	921.4267	921.4192	8.18	0	K.FLENEDR.R

No match to: 1012.5630, 1102.5810, 1115.5790, 1131.5730, 1226.5590, 1252.6040, 1299.6320, 1301.6500, 1307.6580, 1400.6830, 1427.7180, 1467.7700, 1612.7830, 1644.7460, 1815.9260, 1945.9920

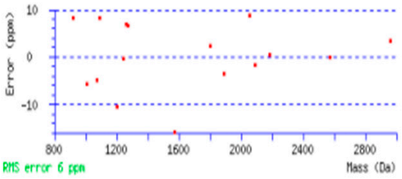

MATRIX SCIENCE MASCOT Search Results

Protein View: APOA1_HUMAN

Apolipoprotein A-I OS=Homo sapiens OX=9606 GN=APOA1 PE=1 SV=1

**Database:**	SwissProt
**Score:**	112
**Expect:**	1.3e-07
**Monoisotopic mass (M_r):**	30759
**Calculated pI:**	5.56
**Taxonomy:**	Homo sapiens

Sequence similarity is available as an NCBI BLAST search of APOA1_HUMAN against nr.

Search parameters

**Enzyme:**	Trypsin: cuts C-term side of KR unless next residue is P.
**Fixed modifications:**	Carbamidomethyl (C)
**Variable modifications:**	Oxidation (M)
**Mass values searched:**	32
**Mass values matched:**	11

Protein sequence coverage: 38%

Matched peptides shown in ***bold red***.

```
  1 MKAAVLTLAV LFLTGSQARH FWQQDEPPQS PWDRVKDLAT VYVDVLKDSG
 51 RDYVSQFEGS ALGKQLNLKL LDNWDSVTST FSKLREQLGP VTQEFWDNLE
101 KETEGLRQEM SKDLEEVKAK VQPYLDDFQK KWQEEMELYR QKVEPLRAEL
151 QEGARQKLHE LQEKLSPLGE EMRDRARAHV DALRTHLAPY SDELRQRLAA
201 RLEALKENGG ARLAEYHAKA TEHLSTLSEK AKPALEDLRQ GLLPVLESFK
251 VSFLSALEEY TKKLNTQ
```

Unformatted sequence string: 267 residues (for pasting into other applications).

Sort by ◉ residue number ○ increasing mass ○ decreasing mass
Show ◉ matched peptides only ○ predicted peptides also

Start - End	Observed	Mr(expt)	Mr(calc)	ppm	M	Peptide
48 - 64	1815.9260	1814.9187	1814.8435	41.4	1	K.DSGRDYVSQFEGSALGK.Q
52 - 64	1400.6830	1399.6757	1399.6620	9.84	0	R.DYVSQFEGSALGK.Q
70 - 83	1612.7830	1611.7757	1611.7781	-1.45	0	K.LLDNWDSVTSTFSK.L
102 - 112	1307.6580	1306.6507	1306.6187	24.5	1	K.ETEGLRQEMSK.D
121 - 130	1252.6040	1251.5967	1251.6136	-13.4	0	K.VQPYLDDFQK.K
131 - 140	1427.7180	1426.7107	1426.6551	39.0	1	K.KWQEEMELYR.Q + Oxidation (M)
132 - 140	1299.6320	1298.6247	1298.5601	49.8	0	K.WQEEMELYR.Q + Oxidation (M)
143 - 155	1467.7700	1466.7627	1466.7841	-14.6	1	K.VEPLRAELQEGAR.Q
176 - 184	1008.4940	1007.4867	1007.5624	-75.1	1	R.ARAHVDALR.T
185 - 195	1301.6500	1300.6427	1300.6411	1.21	0	R.THLAPYSDELR.Q
231 - 239	1012.5630	1011.5557	1011.5713	-15.4	0	K.AKPALEDLR.Q

No match to: 922.4340, 1076.6120, 1090.5770, 1102.5810, 1115.5790, 1131.5730, 1205.6650, 1226.5590, 1247.6020, 1263.6060, 1275.6930, 1576.8160, 1644.7460, 1803.9640, 1891.8490, 1945.9920, 2057.9630, 2090.0920, 2186.0410, 2574.3410, 2963.4160

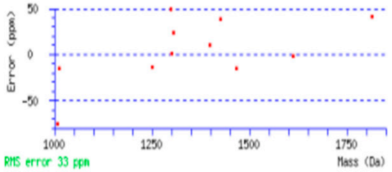

Spot 50

MATRIX SCIENCE Mascot Search Results

```
User          : Claudia Landi
Email         : landi35@unisi.it
Search title  : Siero Endocrinologia
Database      : SwissProt 2021_03 (565254 sequences; 203850821 residues)
Taxonomy      : Homo sapiens (human) (20387 sequences)
Timestamp     : 1 Sep 2021 at 09:57:01 GMT
Top Score     : 329 for Mixture 1, APOA1_HUMAN + A1AT_HUMAN
```

Mascot Score Histogram

Protein score is -10*Log(P), where P is the probability that the observed match is a random event.
Protein scores greater than 56 are significant (p<0.05).

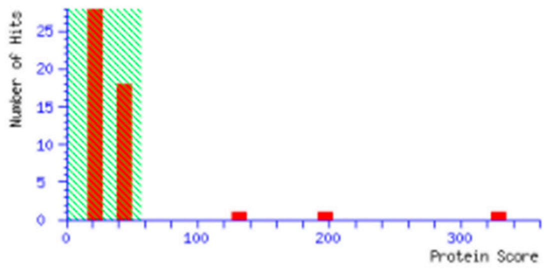

Concise Protein Summary Report

Format As | Concise Protein Summary | Help

Significance threshold p< 0.05 Max. number of hits AUTO

Re-Search All | Search Unmatched

```
1.    Mixture 1    Total score: 329    Expect: 2.6e-29   Matches: 32
      Components (only one family member shown for each component):
      APOA1_HUMAN     Mass: 30759     Score: 202     Expect: 1.3e-16   Matches: 19
      Apolipoprotein A-I OS=Homo sapiens OX=9606 GN=APOA1 PE=1 SV=1
      A1AT_HUMAN     Mass: 46878     Score: 123     Expect: 1e-08   Matches: 14
      Alpha-1-antitrypsin OS=Homo sapiens OX=9606 GN=SERPINA1 PE=1 SV=3
```

MATRIX SCIENCE MASCOT Search Results

Protein View: APOA1_HUMAN

Apolipoprotein A-I OS=Homo sapiens OX=9606 GN=APOA1 PE=1 SV=1

**Database:**	SwissProt
**Score:**	202
**Expect:**	1.3e-16
**Monoisotopic mass (M_r):**	30759
**Calculated pI:**	5.56
**Taxonomy:**	Homo sapiens

Sequence similarity is available as an NCBI BLAST search of APOA1_HUMAN against nr.

Search parameters

**Enzyme:**	Trypsin: cuts C-term side of KR unless next residue is P.
**Fixed modifications:**	Carbamidomethyl (C)
**Variable modifications:**	Oxidation (M)
**Mass values searched:**	36
**Mass values matched:**	19

Protein sequence coverage: 58%

Matched peptides shown in ***bold red***.

```
  1 MKAAVLTLAV LFLTGSQARH FWQQDEPPQS PWDRVKDLAT VYVDVLKDSG
 51 RDYVSQFEGS ALGKQLNLKL LDNWDSVTST FSKLREQLGP VTQEFWDNLE
101 KETEGLRQEM SKDLEEVKAK VQPYLDDFQK KWQEEMELYR QKVEPLRAEL
151 QEGARQKLHE LQEKLSPLGE EMRDRARAHV DALRTHLAPY SDELRQRLAA
201 RLEALKENGG ARLAEYHAKA TEHLSTLSEK AKPALEDLRQ GLLPVLESFK
251 VSFLSALEEY TKKLNTQ
```

Unformatted sequence string: 267 residues (for pasting into other applications).

Sort by ◉ residue number ○ increasing mass ○ decreasing mass
Show ◉ matched peptides only ○ predicted peptides also

Start – End	Observed	Mr(expt)	Mr(calc)	ppm	M	Peptide
35 – 47	1462.8260	1461.8187	1461.8443	-17.5	1	R.VKDLATVYVDVLK.D
37 – 47	1235.6840	1234.6767	1234.6809	-3.39	0	K.DLATVYVDVLK.D
52 – 64	1400.6730	1399.6657	1399.6620	2.69	0	R.DYVSQFEGSALGK.Q
70 – 83	1612.7900	1611.7827	1611.7781	2.89	0	K.LLDNWDSVTSTFSK.L
84 – 101	2202.1150	2201.1077	2201.1117	-1.79	1	K.LREQLGPVTQEFWDNLEK.E
86 – 101	1932.9250	1931.9177	1931.9265	-4.54	0	R.EQLGPVTQEFWDNLEK.E
121 – 130	1252.6270	1251.6197	1251.6136	4.93	0	K.VQPYLDDFQK.K
131 – 140	1427.6660	1426.6587	1426.6551	2.57	1	K.KWQEEMELYR.Q + Oxidation (M)
132 – 140	1283.5900	1282.5827	1282.5652	13.7	0	K.WQEEMELYR.Q
132 – 140	1299.5730	1298.5657	1298.5601	4.33	0	K.WQEEMELYR.Q + Oxidation (M)
165 – 173	1031.5310	1030.5237	1030.5117	11.7	0	K.LSPLGEEMR.D
165 – 173	1047.5190	1046.5117	1046.5066	4.88	0	K.LSPLGEEMR.D + Oxidation (M)
178 – 184	781.4320	780.4247	780.4242	0.64	0	R.AHVDALR.T
185 – 195	1301.6530	1300.6457	1300.6411	3.52	0	R.THLAPYSDELR.Q
202 – 212	1157.6120	1156.6047	1156.6200	-13.2	1	R.LEALKENGGAR.L
213 – 219	831.4260	830.4187	830.4286	-11.9	0	R.LAEYHAK.A
231 – 239	1012.5870	1011.5797	1011.5713	8.37	0	K.AKPALEDLR.Q
240 – 250	1230.7130	1229.7057	1229.7020	3.06	0	R.QGLLPVLESFK.V
251 – 262	1386.7140	1385.7067	1385.7078	-0.79	0	K.VSFLSALEEYTK.K

No match to: 750.3990, 922.4280, 1076.6120, 1090.5770, 1226.5520, 1247.5790, 1263.6340, 1275.6940, 1365.6560, 1576.8540, 1638.8610, 1797.9020, 1803.9660, 1891.8450, 2057.9410, 2090.1140, 2574.3540

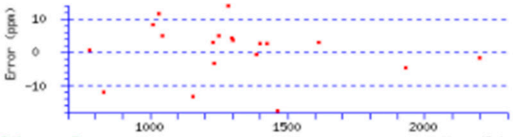

RMS error 7 ppm

MATRIX SCIENCE MASCOT Search Results

Protein View: A1AT_HUMAN

Alpha-1-antitrypsin OS=Homo sapiens OX=9606 GN=SERPINA1 PE=1 SV=3

**Database:**	SwissProt
**Score:**	123
**Expect:**	1e-08
**Monoisotopic mass (M_r):**	46878
**Calculated pI:**	5.37
**Taxonomy:**	Homo sapiens

Sequence similarity is available as an NCBI BLAST search of A1AT_HUMAN against nr.

Search parameters

**Enzyme:**	Trypsin: cuts C-term side of KR unless next residue is P.
**Fixed modifications:**	Carbamidomethyl (C)
**Variable modifications:**	Oxidation (M)
**Mass values searched:**	36
**Mass values matched:**	14

Protein sequence coverage: 34%

Matched peptides shown in ***bold red***.

```
  1 MPSSVSWGIL LLAGLCCLVP VSLAEDPQGD AAQKTDTSHH DQDHPTFNKI
 51 TPNLAEFAFS LYRQLAHQSN STNIFFSPVS IATAFAMLSL GTKADTHDEI
101 LEGLNFNLTE IPEAQIHEGF QELLRTLNQP DSQLQLTTGN GLFLSEGLKL
151 VDKFLEDVKK LYHSEAFTVN FGDTEEAKKQ INDYVEKGTQ GKIVDLVKEL
201 DRDTVFALVN YIFFKGKWER PFEVKDTEEE DFHVDQVTTV KVPMMKRLGM
251 FNIQHCKKLS SWVLLMKYLG NATAIFFLPD EGKLQHLENE LTHDIITKFL
301 ENEDRRSASL HLPKLSITGT YDLKSVLGQL GITKVFSNGA DLSGVTEEAP
351 LKLSKAVHKA VLTIDEKGTE AAGAMFLEAI PMSIPPEVKF NKPFVFLMIE
401 QNTKSPLFMG KVVNPTQK
```

Unformatted sequence string: 418 residues (for pasting into other applications).

Sort by ◉ residue number ○ increasing mass ○ decreasing mass
Show ◉ matched peptides only ○ predicted peptides also

Start – End	Observed	Mr(expt)	Mr(calc)	ppm	M	Peptide
126 – 149	2574.3540	2573.3467	2573.3337	5.06	0	R.TLNQPDSQLQLTTGNGLFLSEGLK.L
154 – 159	750.3990	749.3917	749.3960	-5.64	0	K.FLEDVK.K
161 – 178	2057.9410	2056.9337	2056.9378	-1.98	0	K.LYHSEAFTVNFGDTEEAK.K
188 – 198	1157.6120	1156.6047	1156.6816	-66.4	1	K.GTQGKIVDLVK.E
199 – 215	2090.1140	2089.1067	2089.0884	8.76	1	K.ELDRDTVFALVNYIFFK.G
203 – 215	1576.8540	1575.8467	1575.8337	8.25	0	R.DTVFALVNYIFFK.G
216 – 225	1275.6940	1274.6867	1274.6771	7.52	1	K.GKWERPFEVK.D
218 – 225	1090.5770	1089.5697	1089.5607	8.27	0	K.WERPFEVK.D
226 – 241	1891.8450	1890.8377	1890.8483	-5.62	0	K.DTEEEDFHVDQVTTVK.V
248 – 257	1247.5790	1246.5717	1246.5951	-18.7	0	R.LGMFNIQHCK.K
248 – 257	1263.6340	1262.6267	1262.5900	29.1	0	R.LGMFNIQHCK.K + Oxidation (M)
259 – 267	1076.6120	1075.6047	1075.6100	-4.88	0	K.LSSWVLLMK.Y
284 – 298	1803.9660	1802.9587	1802.9526	3.38	0	K.LQHLENELTHDIITK.F
299 – 305	922.4280	921.4207	921.4192	1.67	0	K.FLENEDR.R

No match to: 781.4320, 831.4260, 1012.5870, 1031.5310, 1047.5190, 1226.5520, 1230.7130, 1235.6840, 1252.6270, 1283.5900, 1299.5730, 1301.6530, 1365.6560, 1386.7140, 1400.6730, 1427.6660, 1462.8260, 1612.7900, 1638.8610, 1797.9020, 1932.9250, 2202.1150

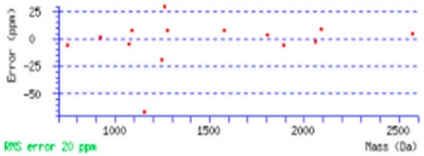

Spot 39

MASCOT Search Results

Protein View: APOA1_HUMAN

Apolipoprotein A-I OS=Homo sapiens OX=9606 GN=APOA1 PE=1 SV=1

**Database:**	SwissProt
**Score:**	100
**Expect:**	2e-06
**Monoisotopic mass (M_r):**	30759
**Calculated pI:**	5.56
**Taxonomy:**	Homo sapiens

Sequence similarity is available as an NCBI BLAST search of APOA1_HUMAN against nr.

Search parameters

**Enzyme:**	Trypsin: cuts C-term side of KR unless next residue is P.
**Fixed modifications:**	Carbamidomethyl (C)
**Variable modifications:**	Oxidation (M)
**Mass values searched:**	27
**Mass values matched:**	10

Protein sequence coverage: 34%

Matched peptides shown in ***bold red***.

```
  1 MKAAVLTLAV LFLTGSQARH FWQQDEPPQS PWDRVKDLAT VYVDVLKDSG
 51 RDYVSQFEGS ALGKQLNLKL LDNWDSVTST FSKLREQLGP VTQEFWDNLE
101 KETEGLRQEM SKDLEEVKAK VQPYLDDFQK KWQEEMELYR QKVEPLRAEL
151 QEGARQKLHE LQEKLSPLGE EMRDRARAHV DALRTHLAPY SDELRQRLAA
201 RLEALKENGG ARLAEYHAKA TEHLSTLSEK AKPALEDLRQ GLLPVLESFK
251 VSFLSALEEY TKKLNTQ
```

Unformatted sequence string: 267 residues (for pasting into other applications).

Sort by ◉ residue number ○ increasing mass ○ decreasing mass
Show ◉ matched peptides only ○ predicted peptides also

Start - End	Observed	Mr(expt)	Mr(calc)	ppm	M	Peptide
102 - 112	1323.6410	1322.6337	1322.6136	15.2	1	K.ETEGLRQEMSK.D + Oxidation (M)
121 - 130	1252.6780	1251.6707	1251.6136	45.7	0	K.VQPYLDDFQK.K
132 - 140	1283.6460	1282.6387	1282.5652	57.3	0	K.WQEEMELYR.Q
185 - 195	1301.6490	1300.6417	1300.6411	0.45	0	R.THLAPYSDELR.Q
213 - 219	831.4350	830.4277	830.4286	-1.08	0	R.LAEYHAK.A
220 - 230	1215.6000	1214.5927	1214.6143	-17.7	0	K.ATEHLSTLSEK.A
231 - 239	1012.5870	1011.5797	1011.5713	8.37	0	K.AKPALEDLR.Q
240 - 250	1230.7070	1229.6997	1229.7020	-1.82	0	R.QGLLPVLESFK.V
251 - 262	1386.7130	1385.7057	1385.7078	-1.52	0	K.VSFLSALEEYTK.K
251 - 263	1514.8010	1513.7937	1513.8028	-5.98	1	K.VSFLSALEEYTKK.L

No match to: 870.5430, 1024.5910, 1040.5850, 1158.6200, 1170.6170, 1179.6030, 1186.6130, 1200.6050, 1213.6810, 1257.6270, 1271.6100, 1313.6610, 1320.6040, 1329.6480, 1339.6050, 1707.7640, 1838.9190

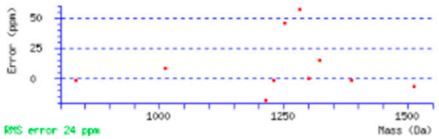

Spot 43

Mascot Search Results

```
User          : Claudia Landi
Email         : landi35@unisi.it
Search title  : Siero Endocrinologia
Database      : SwissProt 2021_03 (565254 sequences; 203850821 residues)
Taxonomy      : Homo sapiens (human) (20387 sequences)
Timestamp     : 1 Sep 2021 at 10:03:53 GMT
Top Score     : 172 for Mixture 1, APOA1_HUMAN + A1AT_HUMAN
```

Mascot Score Histogram

Protein score is -10*Log(P), where P is the probability that the observed match is a random event.
Protein scores greater than 56 are significant (p<0.05).

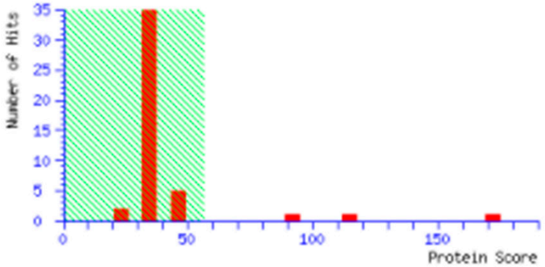

Concise Protein Summary Report

Format As | Concise Protein Summary | Help

Significance threshold p< 0.05 | Max. number of hits AUTO

Re-Search All | Search Unmatched

```
1.   Mixture 1    Total score: 172    Expect: 1.3e-13   Matches: 19
     Components (only one family member shown for each component):
     APOA1_HUMAN    Mass: 30759    Score: 109    Expect: 2.6e-07   Matches: 10
     Apolipoprotein A-I OS=Homo sapiens OX=9606 GN=APOA1 PE=1 SV=1
     A1AT_HUMAN    Mass: 46878    Score: 96    Expect: 4.8e-06   Matches: 10
     Alpha-1-antitrypsin OS=Homo sapiens OX=9606 GN=SERPINA1 PE=1 SV=3
```

MASCOT Search Results

Protein View: APOA1_HUMAN

Apolipoprotein A-I OS=Homo sapiens OX=9606 GN=APOA1 PE=1 SV=1

**Database:**	SwissProt
**Score:**	109
**Expect:**	2.6e-07
**Monoisotopic mass (M_r):**	30759
**Calculated pI:**	5.56
**Taxonomy:**	Homo sapiens

Sequence similarity is available as an NCBI BLAST search of APOA1_HUMAN against nr.

Search parameters

**Enzyme:**	Trypsin: cuts C-term side of KR unless next residue is P.
**Fixed modifications:**	Carbamidomethyl (C)
**Variable modifications:**	Oxidation (M)
**Mass values searched:**	27
**Mass values matched:**	10

Protein sequence coverage: 35%

Matched peptides shown in ***bold red***.

```
  1 MKAAVLTLAV LFLTGSQARH FWQQDEPPQS PWDRVKDLAT VYVDVLKDSG
 51 RDYVSQFEGS ALGKQLNLKL LDNWDSVTST FSKLREQLGP VTQEFWDNLE
101 KETEGLRQEM SKDLEEVKAK VQPYLDDFQK KWQEEMELYR QKVEPLRAEL
151 QEGARQKLHE LQEKLSPLGE EMRDRARAHV DALRTHLAPY SDELRQRLAA
201 RLEALKENGG ARLAEYHAKA TEHLSTLSEK AKPALEDLRQ GLLPVLESFK
251 VSFLSALEEY TKKLNTQ
```

Unformatted sequence string: 267 residues (for pasting into other applications).

Sort by ◉ residue number ○ increasing mass ○ decreasing mass
Show ◉ matched peptides only ○ predicted peptides also

Start – End	Observed	Mr(expt)	Mr(calc)	ppm	M	Peptide
52 – 64	1400.6630	1399.6557	1399.6620	-4.45	0	R.DYVSQFEGSALGK.Q
70 – 83	1612.7840	1611.7767	1611.7781	-0.83	0	K.LLDNWDSVTSTFSK.L
84 – 101	2202.1300	2201.1227	2201.1117	5.02	1	K.LREQLGPVTQEFWDNLEK.E
86 – 101	1932.9250	1931.9177	1931.9265	-4.54	0	R.EQLGPVTQEFWDNLEK.E
102 – 112	1307.6760	1306.6687	1306.6187	38.3	1	K.ETEGLRQEMSK.D
121 – 130	1252.5850	1251.5777	1251.6136	-28.6	0	K.VQPYLDDFQK.K
132 – 140	1299.6130	1298.6057	1298.5601	35.1	0	K.WQEEMELYR.Q + Oxidation (M)
165 – 173	1031.5540	1030.5467	1030.5117	34.0	0	K.LSPLGEEMR.D
165 – 175	1302.6910	1301.6837	1301.6398	33.8	1	K.LSPLGEEMRDR.A
176 – 184	1008.5020	1007.4947	1007.5624	-67.2	1	R.ARAHVDALR.T

No match to: 750.3870, 1090.5730, 1102.5800, 1179.6030, 1205.6650, 1226.5500, 1267.7070, 1275.6920, 1287.6940, 1333.7600, 1576.8280, 1638.8600, 1891.8490, 2057.9450, 2574.3510, 2705.1950, 2963.4090

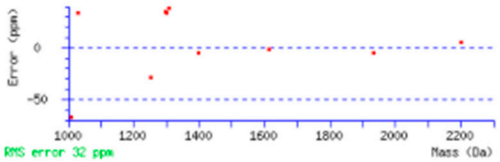

MASCOT Search Results

Protein View: A1AT_HUMAN

Alpha-1-antitrypsin OS=Homo sapiens OX=9606 GN=SERPINA1 PE=1 SV=3

**Database:**	SwissProt
**Score:**	96
**Expect:**	4.8e-06
**Monoisotopic mass (M_r):**	46878
**Calculated pI:**	5.37
**Taxonomy:**	Homo sapiens

Sequence similarity is available as an NCBI BLAST search of A1AT_HUMAN against nr.

Search parameters

**Enzyme:**	Trypsin: cuts C-term side of KR unless next residue is P.
**Fixed modifications:**	Carbamidomethyl (C)
**Variable modifications:**	Oxidation (M)
**Mass values searched:**	27
**Mass values matched:**	10

Protein sequence coverage: 23%

Matched peptides shown in ***bold red***.

```
  1 MPSSVSWGIL LLAGLCCLVP VSLAEDPQGD AAQKTDTSHH DQDHPTFNKI
 51 TPNLAEFAFS LYRQLAHQSN STNIFFSPVS IATAFAMLSL GTKADTHDEI
101 LEGLNFNLTE IPEAQIHEGF QELLRTLNQP DSQLQLTTGN GLFLSEGLKL
151 VDKFLEDVKK LYHSEAFTVN FGDTEEAKKQ INDYVEKGTQ GKIVDLVKEL
201 DRDTVFALVN YIFFKGKWER PFEVKDTEEE DFHVDQVTTV KVPMMKRLGM
251 FNIQHCKKLS SWVLLMKYLG NATAIFFLPD EGKLQHLENE LTHDIITKFL
301 ENEDRRSASL HLPKLSITGT YDLKSVLGQL GITKVFSNGA DLSGVTEEAP
351 LKLSKAVHKA VLTIDEKGTE AAGAMFLEAI PMSIPPEVKF NKPFVFLMIE
401 QNTKSPLFMG KVVNPTQK
```

Unformatted sequence string: 418 residues (for pasting into other applications).

Sort by ◉ residue number ○ increasing mass ○ decreasing mass
Show ◉ matched peptides only ○ predicted peptides also

Start – End	Observed	Mr(expt)	Mr(calc)	ppm	M	Peptide
126 – 149	2574.3510	2573.3437	2573.3337	3.90	0	R.TLNQPDSQLQLTTGNGLFLSEGLK.L
150 – 159	1205.6650	1204.6577	1204.6703	-10.5	1	K.LVDKFLEDVK.K
154 – 159	750.3870	749.3797	749.3960	-21.7	0	K.FLEDVK.K
161 – 178	2057.9450	2056.9377	2056.9378	-0.032	0	K.LYHSEAFTVNFGDTEEAK.K
180 – 187	1008.5020	1007.4947	1007.4924	2.35	0	K.QINDYVEK.G
203 – 215	1576.8280	1575.8207	1575.8337	-8.25	0	R.DTVFALVNYIFFK.G
216 – 225	1275.6920	1274.6847	1274.6771	5.95	1	K.GKWERPFEVK.D
218 – 225	1090.5730	1089.5657	1089.5607	4.60	0	K.WERPFEVK.D
218 – 241	2963.4090	2962.4017	2962.3985	1.09	1	K.WERPFEVKDTEEEDFHVDQVTTVK.V
226 – 241	1891.8490	1890.8417	1890.8483	-3.50	0	K.DTEEEDFHVDQVTTVK.V

No match to: 1031.5540, 1102.5800, 1179.6030, 1226.5500, 1252.5850, 1267.7070, 1287.6940, 1299.6130, 1302.6910, 1307.6760, 1333.7600, 1400.6630, 1612.7840, 1638.8600, 1932.9250, 2202.1300, 2705.1950

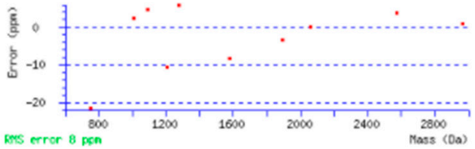

Spot 44

MASCOT Search Results

Protein View: APOA1_HUMAN

Apolipoprotein A-I OS=Homo sapiens OX=9606 GN=APOA1 PE=1 SV=1

**Database:**	SwissProt
**Score:**	199
**Expect:**	2.6e-16
**Monoisotopic mass (M_r):**	30759
**Calculated pI:**	5.56
**Taxonomy:**	Homo sapiens

Sequence similarity is available as an NCBI BLAST search of APOA1_HUMAN against nr.

Search parameters

**Enzyme:**	Trypsin: cuts C-term side of KR unless next residue is P.
**Fixed modifications:**	Carbamidomethyl (C)
**Variable modifications:**	Oxidation (M)
**Mass values searched:**	30
**Mass values matched:**	16

Protein sequence coverage: 41%

Matched peptides shown in ***bold red***.

```
  1 MKAAVLTLAV LFLTGSQARH FWQQDEPPQS PWDRVKDLAT VYVDVLKDSG
 51 RDYVSQFEGS ALGKQLNLKL LDNWDSVTST FSKLREQLGP VTQEFWDNLE
101 KETEGLRQEM SKDLEEVKAK VQPYLDDFQK KWQEEMELYR QKVEPLRAEL
151 QEGARQKLHE LQEKLSPLGE EMRDRARAHV DALRTHLAPY SDELRQRLAA
201 RLEALKENGG ARLAEYHAKA TEHLSTLSEK AKPALEDLRQ GLLPVLESFK
251 VSFLSALEEY TKKLNTQ
```

Unformatted sequence string: 267 residues (for pasting into other applications).

Sort by ◉ residue number ○ increasing mass ○ decreasing mass
Show ◉ matched peptides only ○ predicted peptides also

Start – End	Observed	Mr(expt)	Mr(calc)	ppm	M	Peptide
2 – 19	1858.9370	1857.9297	1858.1040	-93.8	1	M.KAAVLTLAVLFLTGSQAR.H
35 – 47	1462.8300	1461.8227	1461.8443	-14.7	1	R.VKDLATVYVDVLK.D
37 – 51	1650.8540	1649.8467	1649.8625	-9.54	1	K.DLATVYVDVLKDSGR.D
48 – 64	1815.8490	1814.8417	1814.8435	-0.98	1	K.DSGRDYVSQFEGSALGK.Q
52 – 64	1400.6730	1399.6657	1399.6620	2.69	0	R.DYVSQFEGSALGK.Q
70 – 83	1612.7820	1611.7747	1611.7781	-2.07	0	K.LLDNWDSVTSTFSK.L
84 – 101	2202.1230	2201.1157	2201.1117	1.84	1	K.LREQLGPVTQEFWDNLEK.E
86 – 101	1932.9380	1931.9307	1931.9265	2.19	0	R.EQLGPVTQEFWDNLEK.E
86 – 107	2618.2970	2617.2897	2617.2660	9.07	1	R.EQLGPVTQEFWDNLEKETEGLR.Q
102 – 107	704.3150	703.3077	703.3501	-60.2	0	K.ETEGLR.Q
102 – 112	1307.6440	1306.6367	1306.6187	13.8	1	K.ETEGLRQEMSK.D
121 – 130	1252.6260	1251.6187	1251.6136	4.13	0	K.VQPYLDDFQK.K
121 – 131	1380.7160	1379.7087	1379.7085	0.15	1	K.VQPYLDDFQKK.W
131 – 140	1427.6730	1426.6657	1426.6551	7.47	1	K.KWQEEMELYR.Q + Oxidation (M)
132 – 140	1283.5760	1282.5687	1282.5652	2.76	0	K.WQEEMELYR.Q
132 – 140	1299.5760	1298.5687	1298.5601	6.64	0	K.WQEEMELYR.Q + Oxidation (M)

No match to: 1093.5130, 1158.5930, 1165.5660, 1226.5600, 1277.7120, 1311.5790, 1314.6870, 1638.8420, 1644.7430, 1669.8110, 1683.8170, 1716.8510, 2717.1430, 2887.5070

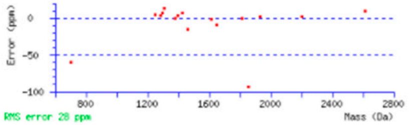

RMS error 28 ppm
